# Challenges in the Timely Diagnosis of Behcet’s Disease

**DOI:** 10.3390/life13051157

**Published:** 2023-05-11

**Authors:** Fadi Hassan, Helana Jeries, Mohammad E. Naffaa

**Affiliations:** 1 Rheumatology Unit, Galilee Medical Center, Naharyia 2210001, Israel; 2 Azrieli Faculty of Medicine, Bar-Ilan University, Safed 1311502, Israel

**Keywords:** Behcet’s disease, criteria, diagnosis, international study group, the international criteria for Behcet’s disease

## Abstract

Behcet’s disease (BD) is a chronic, multi-systemic inflammatory disorder mainly characterized by recurrent oral and genital ulcers, skin lesions, and uveitis. As no pathognomonic laboratory test exists for BD, the diagnosis relies solely on clinical features. Over the years, great efforts have been invested in creating clinical diagnostic and classification criteria. The international study group criteria introduced in 1990 were the first true multinational set of criteria. Despite improving the ability to diagnose BD, these criteria still have limitations, including the inability to diagnose patients presenting without oral ulcers or presenting with rare manifestations of the disease. This led to the introduction of the international criteria for BD in 2013, which improved the sensitivity with minimal compromise on specificity. Despite the efforts made and as our understanding of the clinical manifestations of BD and genetic pathogenesis continue to evolve, efforts should be made to further enhance the currently accepted international classification criteria, perhaps by incorporating genetic testing (e.g., family history or HLA typing) as well as ethnic group-specific features.

## 1. Introduction

Behcet’s disease (BD) is a chronic, multi-systemic inflammatory disorder mainly characterized by recurrent oral and genital ulcers, skin lesions, and uveitis [1]. Other systems, including central nervous, cardiovascular, gastrointestinal, and musculoskeletal systems, may be involved with varying prevalence [2,3,4,5,6,7]. The disease has a unique geographic distribution that follows the ancient Silk Road, extending from Japan to the Mediterranean area, with its highest prevalence in Turkey, followed by Israel and Iran [8,9,10,11]. Certain manifestations are more prominent in some geographic areas, and others may be more common in a specific gender [12]. For example, gastrointestinal manifestation is more common in the Far East than in the Mediterranean area, while vascular, ocular, and neurologic manifestations are more common in male patients [12,13,14]. As no pathognomonic laboratory test exists for BD, the diagnosis relies on clinical features. Over the years, great efforts have been made to create clinical diagnostic and classification criteria with optimal sensitivity and specificity. Several sets of criteria were suggested, including, but not limited to, Curth, Mason and Barnes, Hewett (revised), Japan (original and revised), Hubaulat and Hamza, O’Duffy, Cheng and Zhang, Dilsen (original and revised), International Study Group (ISG), Iran Traditional, Korea and International Criteria for Behcet’s Disease (ICBD) (Table 1) [15,16].

It is of paramount importance at this point to differentiate between diagnostic and classification criteria, as they are sometimes used interchangeably. While classification criteria aim to produce a homogenous group of patients mainly for clinical trials and, therefore, may miss patients with rare or uncommon manifestations, diagnostic criteria aim to include as many patients as possible, including patients at the extremes of the disease’s spectrum.

The current review will focus mainly on the ISG and the ICBD sets of criteria, as they are the most commonly used and because they constitute a true attempt to build the criteria on the basis of multinational collaboration. Further, this review will discuss some challenges in the timely diagnosis of BD based on our ongoing experience with the unique Druze population in northern Israel.

## 2. The International Study Group Criteria

The International Study Group (ISG) criteria for BD were introduced in 1990 as the first true multinational set of criteria [26]. These criteria were the most commonly used until the appearance of the ICBD criteria in 2013, though they are still used today. The ISG criteria were based on 914 patients diagnosed with BD, of whom 366 patients were from Iran, 285 from Turkey, 141 from Japan, 50 from Tunisia, 21 from the UK, 14 from the USA, and 9 from France [26]. BD patients were compared to controls from the same centers. The diagnosis of BD in these patients was based on the decision of an experienced physician and according to the diagnostic criteria with which he was most familiar. Interestingly, 28 (3%) patients were excluded because they did not have oral ulceration and, therefore, were not included in the analysis, although the study group members were aware that, rarely, patients with BD may present without oral ulcerations [26]. The data for each participating patient and control were recorded for the presence or absence of all features of the disease included in all sets of diagnostic criteria available at that time. For each patient, every feature was recorded as currently present or evident in the past, as well as whether it was observed by the physician or the patient; in the final analysis, however, no discrimination was made between past or present features. Additionally, each patient underwent a pathergy test. Data from a randomly chosen 60% sample of the total study population of BD were used to calculate the expected weight of evidence for each individual feature that was derived from the log-likelihood ratio for the presence or absence of the feature and its prevalence in BD. Genital ulcerations, ocular lesions, positive pathergy test, folliculitis, erythema nodosum, and the combination of folliculitis and erythema nodosum, all showed good discrimination for BD, while none of the other features showed useful diagnostic value, namely subcutaneous thrombophlebitis, deep vein thrombosis, epididymitis, arterial occlusion and/or aneurysm, CNS involvement, arthralgia, arthritis, gastrointestinal features, and, surprisingly enough, family history [26]. Furthermore, the presence or absence of highly suggestive features of BD, such as arterial aneurysm, CNS involvement, or family history, did not contribute to the confirmation or ruling out of the diagnosis. A new set of criteria, the ISG criteria, was launched and required the presence of recurrent oral ulcers as an obligatory entry criterion in addition to two of the following four criteria—genital ulcers, ocular involvement, positive pathergy test, and folliculitis or erythema nodosum (Table 2) [26]. These criteria were slightly less sensitive than other sets of criteria used at that time but more specific for the clinical diagnosis of BD [15,16].

Although the ISG criteria represented an upgrade of the commonly-used sets of criteria at that time and a real attempt to create a new set of criteria based on a multinational representation, some concerns were raised. First, about 3% of patients with BD will not be timely diagnosed with the disease because they do not present with recurrent oral ulcers even though they have an almost pathognomonic presentation of the disease with pulmonary artery aneurysms which, in the appropriate setting, are almost exclusively due to BD [26]. Although the suspicion of BD will arise very early if the treating physician is experienced with BD, the diagnosis is likely to be missed or at least delayed by a treating physician less familiar or unfamiliar with BD.

Second, even when the patient presents with recurrent oral ulcerations and another rare manifestation of the disease (such as recurrent sudden sensory neural hearing loss) but without other criterion features, the patient may not be diagnosed with BD in a timely manner until other criteria features of BD become evident [26]. When a young male Druze patient in northern Israel, a population with a very high prevalence of BD, presented with recurrent oral ulcerations and recurrent sudden sensory neural hearing loss, even though he did not meet the criteria at that time, the leading and most probable diagnosis is still BD until proven otherwise [10]. Interestingly, in our new and small but rapidly increasing cohort of patients with BD in northern Israel, we have already identified five families with at least two family members each affected with BD. This example highlights the notion that pretest probability, as well as family history, should be taken into account when considering BD diagnosis.

Third, although the study group tried to include patients from several countries, some endemic countries with a high prevalence of BD (Israel and Saudi Arabia, for example) were not included [8,9,10,11,26]. The prevalence of BD in Israel varies according to the specific ethnic group, with the highest prevalence among the Druze in northern Israel, followed by Arab Muslims, according to a study by Krause et al. (about 146 cases per 100,000 and 26 cases per 100,000, respectively) [10,11]. The Druze ethnic group in Israel is located almost exclusively in northern Israel and is characterized by high rates of intra-family marriages, which undoubtedly contributes to the high prevalence of the disease, thus highlighting the importance of the genetic factor in BD [10,32,33]. A good example is a hypothetical case of a young male Druze patient who presents with deep vein thrombosis (DVT) and has a family history of BD, as opposed to a young male from a northern European background who presents with DVT. While in the first case, the most probable diagnosis is BD and the main treatment is immunosuppression, in the second case, other diagnoses are more probable than BD and the principal treatment is anticoagulation [34]. Therefore, we believe that a known family history in ethnic groups where BD is endemic increases the likelihood of a BD diagnosis and should be taken into account when evaluating a patient from such a population, even when the criteria are not fully met.

## 3. The International Criteria for Behcet’s Disease

Although the ISG criteria improved the sensitivity and specificity of the commonly-used sets of criteria at that time, the limitations mentioned here have led to continued efforts to develop more comprehensive criteria without compromising specificity. Thus, in 2013, the International Society of Behcet’s Disease (ISBD) initiated a collaborative multinational study to examine the sensitivity and specificity of the ISG criteria in a large multinational cohort and, depending on the results, whether a new set of criteria should be created [15,31,35,36]. While the specificity of ISG criteria in this multinational cohort was 95.9%, the sensitivity was relatively low (81.2%), showing that the need for a new set of criteria was desirable. The “new” criteria were based on a total of 2556 patients with BD and 1163 controls from 27 countries worldwide, including countries with a high prevalence of BD, such as Israel and Saudi Arabia, which had not been included in the ISG (Austria, Azerbaijan, China, Egypt, France, Germany, Greece, India, Iran, Iraq, Israel, Italy, Japan, Jordan, Libya, Morocco, Pakistan, Portugal, Russia, Saudi Arabia, Singapore, Spain, Taiwan, Thailand, Tunisia, Turkey, and USA) [31]. The patients and the controls were recruited based on the presenting symptoms before treatment, and the diagnosis of BD was made on a clinical basis by an expert physician in each center, who was advised not to follow any other diagnostic algorithm for the final diagnosis [31]. Control patients were selected from patients with other final diagnoses mimicking BD or patients having at least one major sign attributable to BD. Complete clinical data were required for all patients except for pathergy testing, HLA typing, and family history [31]. The full dataset was divided randomly into two sets: training and validation sets, stratified by country and case/control status. The training set was used for the initial comparison of the ISG criteria with other criteria as well as for the development of the revised “new” criteria, and the validation set was used to compare the “new” criteria with the other criteria. To ensure that only variables with discriminant utility be included in the final model, a p-value of less than 0.01 for the test of association with the disease was used. Notably, variables that were not routinely collected in all patients (e.g., pathergy test, HLA typing, and family history) were not considered for inclusion in the development of the new criteria. Importantly, an attempt to limit the influence of data from any one country was made by allowing each country to contribute no more than 10% of the information for cases and no more than 10% of the information for controls [31]. A leave-one-country-out-at-a-time cross-validation approach along with inspection of receiver operator characteristic (ROC) curves of estimated sensitivity vs. specificity were used to assess the utility of adding more variables. By using this method, a number of schemes/scores were revealed, and the project’s leaders decided on the most appropriate scheme that would best serve the predefined goals of this collaboration. Moreover, the additional value of adding the result of the pathergy test, when available, was examined.

The above-mentioned process resulted in the emergence of the ICBD criteria (Table 3) with relatively high sensitivity and specificity, 93.9% and 92.1%, respectively, in the training set and 94.8% and 90.5%, respectively, in the validation set [31]. The addition of the result of the pathergy test, when available, improved sensitivity with minimal negative effect on specificity [31]. It is worth noting that, contrary to the ISG criteria, where recurrent oral ulcers was an obligatory entry criterion, in the ICBD criteria, a patient can be diagnosed with BD even without recurrent oral ulcers, which may appear later in the disease course in some patients (Table 3). Second, the ICBD included neurologic and vascular manifestations in its criteria in contrast to the ISG criteria (Table 3).

Although the ICBD improved the sensitivity compared to the ISG (94.8% vs. 81.2%, respectively) with relatively similar specificity (90.5% vs. 95.5%, respectively), some concerns were raised. First, according to the “new” ICBD criteria, any patient presenting with recurrent oral and genital ulcers receives four points and thus qualifies for BD diagnosis. However, the mere presence of recurrent oral and genital ulcers, a condition known as bipolar aphthosis (or complex aphthosis), does have a rather broad range of differential diagnoses besides BD, including idiopathic complex aphthosis, drug eruptions, Crohn’s disease, reactive arthritis, gluten-sensitive enteropathy, Sweet syndrome, erythema multiforme, viral infections such as herpes and cytomegalovirus infections, mevalonate kinase disease, haploinsufficiency of A20, deficiency of ADA2, and several immunodeficiencies [37,38,39]. An experienced physician caring for patients with BD will probably succeed in differentiating those conditions from BD. However, a physician unfamiliar with BD may incorrectly diagnose BD and administer unnecessary treatment.

**Figure 1:**
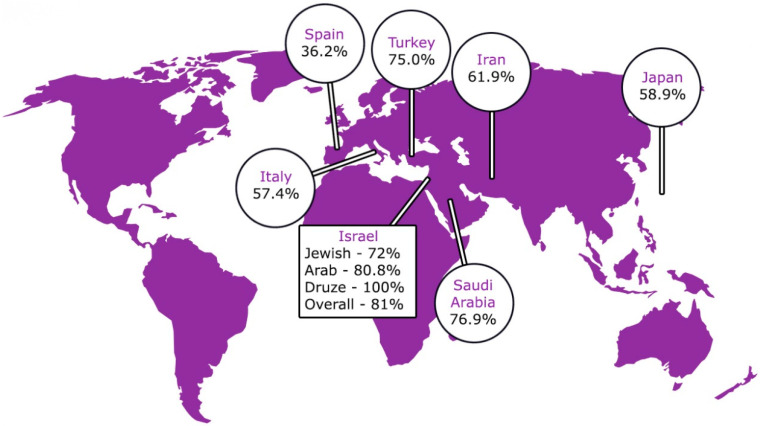
Prevalence of HLA-B51 positivity in Behcet’s patients worldwide.

Second, the unique geographic distribution of BD across the ancient “Silk Route” may reflect a genetic background predisposition as having a key role in the pathogenesis of BD. In recent years, several genomic studies have identified multiple susceptibility genes, and among them, HLA-B51 is considered the strongest, with some studies attributing 32–52% of the population’s risk of developing BD to this allele [40,41,42,43,44,45,46]. Not surprisingly, HLA-B51 positivity is much higher in regions endemic to BD, with frequency ranging from 50 to 80% among BD patients in these areas (Figure 1) [10,11,47]. Takeno et al. showed a very high frequency of HLAB51 positivity in regions endemic to BD, with a prevalence of 76.9% and 58.9% in Saudi Arabia and Japan, respectively (Figure 1) [10,11,47]. These data highlight the genetic factor in the pathogenesis of BD and could suggest that the inclusion of HLA typing and family history may further improve the “new” ICBD diagnostic criteria.

## 4. Looking to the Future

The ISG and ICBD criteria improved the ability to diagnose Behcet’s disease at an early stage, but both criteria still have their own limitations, as detailed above. We postulate that both sets of criteria can still be improved based on the following.

First, as previously mentioned, BD disease is a multifactorial inflammatory disorder with a significant genetic component, supported mainly by its familial aggregation and increased prevalence of positivity to HLAB51, as well as other HLA alleles among BD patients [40,41,42,43,44,45,46]. BD is common in the Israeli population, especially among non-Ashkenazi Jews, Muslim Arabs, and Druze [10,11]. The Druze population is a population with a unique historical, religious, and demographic structure. The contemporary Druze population resides as a minority, mainly in four Middle Eastern countries—Syria, Lebanon, Israel, and Jordan. The Israeli Druze population, estimated at 150,000, is located mainly in northern Israel in the Galilee and the Carmel regions; many are being treated in our hospital. This population is genetically unique due to the extremely low frequency of admixture with populations other than Druze; in some instances, the Druze custom strongly favors marriage within the same village or the same geographical area [32,33]. Furthermore, unlike other monotheistic religions, the Druze religion is strictly closed to new adherents, thus, further preventing admixture with other populations [32]. This unique social structure has turned the Druze into a genetically-isolated population. In our own experience, the Druze population shows a very high prevalence of BD, estimated at 146 per 100,000 according to one study (which we believe is an underestimation of the true prevalence, as it did not include all medical centers taking BD in northern Israel), probably lending this population the highest prevalence of BD in the world with the exception of Turkey, where BD is estimated at 400 per 100,000 [10]. The social structure of the genetically-isolated Druze society, along with the very high prevalence of BD, emphasizes the importance of the genetic component in the pathogenesis of BD. Interestingly, in our new and small but rapidly increasing cohort of patients with BD in northern Israel, we have already identified five Druze families with at least two family members each affected with BD, thus highlighting the role of the genetic component in the pathogenesis of BD.

Secondly, although previous studies showed that BD has no clinical phenotypic differences among ethnic groups, recent studies have revealed that the clinical presentation of BD varies considerably between ethnic groups and countries [12,48,49]. Although recurrent mouth ulcers were found almost universally, other manifestations are more common in certain ethnic populations. For instance, while there are no significant differences regarding neurological features of BD between Caucasians and those of Middle Eastern origin, a previous study showed that the frequency of neurological features was far more common in Caucasians compared to Middle Eastern patients [50]. Moreover, the frequency of seizures was found to be sevenfold higher in Caucasians than in the Turkish population [50]. In another study, genital ulcers, especially those occurring close to the anal sphincter, were more common in Western countries compared to other populations [51]. It has been shown that gastrointestinal manifestations are more common in the Far East than in the Mediterranean area [12]. A further example is the pathergy reaction, which is highly sensitive and specific for BD in patients from the Silk Road but may be negative in patients from Western countries [48]. Genetic factors may also show variability, as demonstrated by the fact that HLA-B51 positivity is higher in certain countries compared to others. In a study performed in Israel, all Druze patients were HLA-B5-positive compared to 80.8% of the Arab Muslim patients and 72.0% of the Jewish patients with BD, according to a study by Krause et al. [10].

Both sets of criteria, the ISG and ICBD, have improved sensitivity, specificity, and standardization when diagnossing patients with BD. However, it is imperative to remember that both criteria are truly and genuinely representative of the source populations and dataset on which they were built. Thus, despite the significant improvement presented by the two sets of criteria, based on our own experience and especially with a population such as the highly homogenous Druze population in our region, it is still very important to consider the genetic component and family history as part of the considerations for a BD diagnosis, and especially in patients presenting with an uncommon feature of BD. These factors further increase the complexity of making one set of truly universal criteria suitable to diagnose all BD patients in all ethnic backgrounds with adequate sensitivity and specificity. In the current era of personalized medicine, efforts should be made to determine whether one true set of criteria can actually diagnose all cases of BD with adequate sensitivity and specificity. Perhaps family history, HLA typing, or ethnic group-specific features should be incorporated into the current list of criteria. Further research is needed in this area.

## 5. Conclusions

In conclusion, the introduction of ISG and ICBD criteria improved the ability to diagnose BD at an early stage, but both criteria still have their own limitations. As our understanding of this disease and its pathogenesis continues to evolve, efforts should be made to further enhance the currently-accepted international classification criteria, perhaps by incorporating genetic testing (e.g., HLA typing) as well as ethnic group-specific features.

## Figures and Tables

**Table 1:** Sensitivity and specificity of different criteria sets.

Criteria	Sensitivity	Specificity
Curth [17]	99%	82%
Mason [18]	82%	95%
Hewitt [19]	57%	96%
Japan Original [20]	88%	92%
Hubault and Hamza [21]	58%	97%
O’Duffy [22]	88%	90%
Cheng and Zhang [23]	98%	83%
Dilsen (original) [24]	88%	91%
Japan (revised) [25]	91%	91%
International Study Group [26]	85%	96%
Iran traditional [27]	90%	92%
Iran—Classification Tree [28]	96%	90%
Dilsen revised [29]	87%	96%
Korea [30]	92%	92%
International Criteria for Behcet’s Disease [31]	95%	91%

Sensitivity and specificity are based on the international study group cohort validation set.

**Table 2:** The International Study Group Criteria.

For a clinical diagnosis of Behcet’s disease, the patient must have recurrent oral ulceration (minor [<10 mm] or major aphthous [>10 mm], or herpetiform ulcers observed by the physician or reliably described by the patient, which recurred at least three times over a 12-month period) in addition to at least two of the following additional findings in the absence of any other clinical explanation:
**Recurrent genital ulceration:** Aphthous ulceration or scarring observed by the physician or reliably described by the patient;
2. **Ocular lesions:** Anterior or posterior uveitis or cells in the vitreous body on slit-lamp examination or retinal vasculitis detected by an ophthalmologist;
3. **Skin lesions:** Erythema nodosum, pseudofolliculitis, papulopustular lesions, or acneiform nodules not related to glucocorticoid treatment or adolescence;
4. **Positive pathergy test:** Test interpreted as positive by the physician at 24–48 h.
*Sensitivity of 91–95% and a specificity of 85–98% in the international study group cohort compared to 81.2% sensitivity and 95.9% specificity in a multinational cohort.*

**Table 3:** International Criteria for Behcet’s Disease (2013).

Point score system: scoring 4 indicates diagnosis of Behcet’s disease	
**Sign or Symptom**	**Points**
Ocular lesions	2
Genital ulcers	2
Oral ulcers	2
Skin lesions	1
Neurologic manifestations	1
Vascular manifestations	1
Positive pathergy test result	1
*Sensitivity of 93.9–94.8% and a specificity of 90.5–92.1% in the international criteria for Behcet’s disease cohort.*	

## Data Availability

Not applicable.
